# Nonsurgical management of temporomandibular joint autoimmune disorders

**DOI:** 10.3934/publichealth.2019.4.554

**Published:** 2019-12-12

**Authors:** Ehsan Shoohanizad, Ata Garajei, Aida Enamzadeh, Amir Yari

**Affiliations:** 1 Assistant Professor, Department of Oral and Maxillofacial Surgery, Taleghani Hospital, Kermanshah University of Medical Sciences, Kermanshah, Iran; 2 Associate Professor, Department of Oral and Maxillofacial Surgery, School of Dentistry and Department of Head and Neck Surgical Oncology and Reconstructive Surgery, The Cancer Institute, School of Medicine, Tehran University of Medical Sciences, Tehran, Iran; 3 DMD, Private Practice, Tehran University of Medical Sciences, Tehran, Iran

**Keywords:** temporomandibular joint disorders, systemic lupus erythematosus, rheumatoid arthritis, pain, nonsurgical management, autoimmune diseases

## Abstract

**Introduction:**

Temporomandibular disorders (TMD) are observed in a number of autoimmune diseases but limited studies have assessed the effect of autoimmune diseases on TMD. Therefore, the present review article was conducted to determine the effect of autoimmune diseases on TMD.

**Methods:**

International databases, including Web of Sciences, PubMed and Scopus, were searched in order to find related articles. The search key words were; temporomandibular joint (TMJ) autoimmune disorders, TMJ, TMD, medical therapy and non-invasive, local and systemic management. Published articles from June, 2010 to June, 2019 were included in the review.

**Results:**

A total of 11 related articles including rheumatoid arthritis (RA), lupus erythematosus and systemic sclerosis were found. All articles noted that TMJ has unique features that distinguishes it from other human body joints. Cases of TMJ injury and TMD require specific treatments. Therefore, early diagnosis of TMD is essential. It was also mentioned in the articles that the collagen-induced arthritis (CIA) method is a suitable method for investigating TMD and its relationship with RA. Treatment methods included oral steroids, Disease-modifying antirheumatic drugs, nonsteroidal anti-inflammatory drugs, methotrexate 75 mg, and combination therapy with methotrexate.

**Conclusion:**

Based on the results of this study, TMD exists in some autoimmune diseases, including RA, lupus erythematosus and systemic sclerosis. Therefore, there should be an interdisciplinary collaboration between physicians and dentists in order to choose the best conservative treatment and medication therapy for TMD to reduce the progression and pain associated with this type of disorder.

## 1. Introduction

Temporomandibular joint (TMJ) has a particular performance. The chronological changes in TMJ are important issues in surgery and radiology [1],[2]. Due to limited knowledge regarding the anatomy, function, and physiological changes of TMJ, numerous diagnostic errors have been observed for temporomandibular diseases (TMDs) [3]. Various conditions, including osteoarthritis and joint disk disorders, can affect TMJ and cause skeletal deformity, malocclusion, and masticatory system dysfunction [4]. The problems of the masticatory system, including TMJ, muscular and dental system and the supporting bones, are called TMD [5]. The prevalence of TMD in adults, ranges from 40% to 70%, and is 16% in children with deciduous dentition and 90% in people with mixed dental systems [6]–[8]. The prevalence, causes and factors affecting TMD, the signs and clinical symptoms vary according to age, race, geographical location and time of assessment [9],[10]. According to various studies, the prevalence of TMD in the international community and among Iranians ranged between 10% and 91% [11],[12]. TMD is generally reported to be 1.5 to 2 times more common in women compared to men and this difference is attributed to behavioral, hormonal, anatomical and psychological factors [13],[14]. TMD is observed in a variety of autoimmune diseases, including rheumatism and osteoarthritis. However, limited studies have been performed to assess the effects of autoimmune diseases on TMD. In a study by Movahedian et al. TMD was investigated in patients with RA. In this study the clinical symptoms of TMD were assessed in 80 cases with definitive diagnosis of RA [15]. In another study by Matos autoimmune diseases were found to be an etiologic factor for TMD [16]. Based on the above-mentioned and limited specialized studies about the effects of autoimmune diseases on TMD, this review study was performed in order to determine the effect of autoimmune diseases in TMD.

## 2 Methods

This narrative review was performed to determine the effect of autoimmune diseases on TMD. Various key words, including temporomandibular joint autoimmune disorders, TMJ, TMD, Systemic management, medical therapy and non-invasive local and systemic management, were used to identify related articles. Articles that were published from June, 2010 to June, 2019 in international databases, including Web of Sciences, PubMed and Scopus, were included in the search. At first, the titles of the identified articles were investigated based on thematic communication. In the next phase, the abstracts were evaluated regarding the relevance to the intended purpose. Then identified articles were included in this study. Articles without thematic connection, method, full text and articles presented at conferences, duplicate articles and articles published in a language other than English were excluded from this study.

The information of the articles was reviewed based on the inclusion and exclusion criteria by two reviewers independently. Finally, both reviewers summarized the information. At first the collected data was summarized into categories, including definitions of TMJ, TMD related factors, signs and symptoms of TMD, TMD diagnostic methods, systemic management and effect of autoimmune diseases on TMD. Finally, the results were summarized in Table 1.

## 3. Results

A total of 78 articles were found in the literature search, among which 11 articles were found to be related to the effect of autoimmune diseases on TMD.

### 3.1. Definition of TMJ

TMJ acts like a sliding hinge and attaches the jaw bone to the skull [17]. In other words, TMJ is one of the most used and most important joints in human body, which is comprised of mandibular condyle and glenoid fossa, articular eminence, and articular disc [18],[19]. At the time of mouth opening, the mandibular condyle bears a complex movement. This movement consists of two parts; the rotational and translational movement [20],[21]. In the rotational movement, the upper surface of the condyle rotates against the lower surface of disc and in the translational movement, condyle moves downward and forward and simultaneously moves the disc forward so that the central thin part of the disc is placed between the head of the condyle and the prominence of the joint [21],[22].

In most people, in the maximal mouth opening, Condyle goes down and forward to the prominent peaks and when the mandibul is closed, the disc returns to the mandibular cavity with condyle [23]. There is a possibility of various abnormalities to occur in TMJ, including developmental anomalies of the TMJ, subluxation, soft tissue disorders, remodeling, arthritis, trauma and tumors. The complications of these abnormalities include facial asymmetry, pain and swelling, as well as problems in occlusion, muscle cramps and muscle spasms that finally lead to difficulties in daily routines. Even with the continuation of this situation, there is a possibility of psychological complications, including depression, that interfere with the normal course of life of an individual. TMDs can cause pain in the jaw joint and muscles controlling jaw movements. Identification of the exact causes of TMD disorders is often difficult [20], [24].

### 3.2. Risk factors for TMD

Based on the attitude of many researchers, TMD is multifactorial and complex [25],[26]. But the most important causes of this disease are categorized into acquired factors, hereditary factors and other factors. The acquired factors that affect TMD include; infections, injuries, Iatrogenic (surgery, radiation therapy), habits, tumors, and Idiopathic. The hereditary factors that affect TMD include; Hemifacial microsomia, Hemifacial atrophy, Rheumatoid arthritis (RA) and Ankylosis. Furthermore, other factors that may cause TMD include; muscle spasms, inappropriate occlusal contact, stress, systemic diseases and immunological factors. Besides, some researchers reported the association of TMD with risk factors, including distress, depression, oral habits, unsafe socioeconomic status and genetic backgrounds [27],[28].

### 3.3. Signs and symptoms of TMD

In order to identify signs and symptoms of TMD, a complete medical and dentistry history should be primarily obtained from the patient to detect any inherited or acquired disorder. furthermore, history of trauma and pain should be considered. Then, the clinical examination of the patients begins with the palpation of Temporal muscle, superficial and deep part of masseter muscle, lateral pterygoid, sternocleidomastoid and upper trapezius muscles, occipital bone and cervical muscles [29],[30]. Then, TMJ is palpated in open and closed mouth positions, side movements with focus on pain during the examination and the presence of sound in the joints, as well as Deviation (initial deviation on mouth opening path and return of the jaw to midline at the end) and Deflection (the permanent deviation of the jaw to the end of the mouth opening path) as well as range of protrusive movement and occlusion [31]. The most common signs and symptoms of TMD are pain in palpation of the masticatory muscles, muscular dysfunction, joint sounds, headache, disorders and deviation in jaw movements and mouth opening, dental attrition and occlusal interference [32],[33]. It should be noted that the incidence of TMD increases with the increase in age [32]. Evidently, children have difficulty in describing and localizing pain. Therefore, association of signs and symptoms is not clear in children [34].

### 3.4. Diagnostic methods for TMD

The initial evaluation of TMD is based on a clinical examination of the masticatory muscles. However, several studies have shown that clinical examination is unreliable in many cases [35],[36]. Imaging is an important diagnostic component at determination and interpretation of TMJ diseases. TMD diagnostic imaging techniques include; arthrography, computed tomography and magnetic resonance imaging (MRI). Simple radiography, including Transcranial, proprietary panoramic imaging, and tomography are more frequently used today compared to other methods. Commonly the first TMJ-specific imaging method used in survey of a patient with TMD related complaints, is panoramic imaging. The advantages of panoramic imaging is due to its two-dimensional and the effect of positioning of the head of the patient on image. The advantages include; low-dose radiation, availability, ease of use and low cost. But the anatomical superimposition reduces the value of panoramic images [37],[38]. Computed tomography allows better observation of the bone structures, but high dose of radiation and high cost are the disadvantages of CT scan [39]. Nowadays, CBCT is used in the field of head and neck. The advantages of CBCT include; low radiation dose and higher resolution compared to CT-scan. The CT-scan application is limited in the evaluation of TMD [40]. The cone beam tomography (CBCT) allows the structural examination of the bones, joint space, the dynamic three dimensional function of joint without superimposition and deformation [41].

### 3.5. Non-surgical management

Treatment for TMD is implemented in two distinct phases. The first phase involves patient training for reducing anxiety, behavioral changes, pharmacotherapy, physiotherapy and splint therapy. The second phase involves performing occlusal adjustment, fixed prosthetics, restorative treatments, dental restoration, orthodontics or orthognathic surgeries. A topic called Biofeedback has been suggested in the non-pharmacological treatments to reduce anxiety through behavioral and Biofeedback treatments in both short and long-term. Biofeedback is also more effective in pain management in patients with TMD compared to conventional treatments [42].

Behavioral modifications, including stress reduction, sleep hygiene and avoiding abnormal habits (e.g. clenching, chew pencil or ice) and avoiding severe jaw movements (e.g. opening mouth too much while yawning, teeth brushing) are advised to patients with TMD [43].

Physiotherapy involves the use of TENS, ultrasound and hot pack. Arthroscopy is sometimes helpful to remove intruder connectors inside the joints and cleaning joint through arthrocentesis. On the other hand, there are evidence based researches regarding the effects of occlusal splints. Short-term studies have also shown that the use of the appliance has been effective in reducing signs and symptoms of TMD disorders [44],[45]. Different materials and designs of oral appliances have been developed based on some theories about their functional mechanism. However, the effectiveness of these appliances in the treatment of joint pains and their long-term treatment results has been questioned by some researchers. In a study by van Grootel, physiotherapy massage was found to be a suitable method for pain relief in TMD [46]. In the study by Seifi et al, TENS or Low-Level laser therapy was found to improve TMD symptoms [47]. Pharmacotherapies in TMD include administration of analgesics, sedatives, relaxants and corticosteroids. Dentists may prescribe higher doses of non-steroid anti-inflammatory drugs (NSAIDs), if needed, to reduce pain or swelling. Muscle relaxant medications are recommended to calm the jaw if clenching or bruxism are seen. Anti-anxiety drugs that may lead to TMJ relaxation by reducing stress are also prescribed. NSAIDs, opioids, corticosteroids, anxiolytics, muscle relaxants, antidepressants, anticonvulsants and benzodiazepines should be used in specific doses to be effective [48]. Each of the treatment processes will be described in the following paragraphs.

Only 5% to 10% of patients with TMD need treatment and TMD symptoms reduce spontaneously in 40% of patients. In a long follow up study, 50% to 90% of patients reported a period of pain relief during the course of follow up [49],[50].

The first treatment goal is to manage, reduce and eliminate pain. At first, conservative treatments are prescribed. Conservative treatment may lead to the resolution of symptoms in over 80% of patients. Conservative interventions include resting the jaw by using a soft diet, avoiding excessive opening of the mouth, physiotherapy and the use of NSAIDs, that can be administered topically, and use of soft occlusal appliances made by the dentists. Effective management of the disease with disease-modifying anti rheumatic drugs (DMARDs) is vital for patients [51].

There is no evidence that long-term physiotherapy is beneficial, but due to the safety of physiotherapy, it can be used in short-term management of patients, post arthroscopy or after open surgery. Topical NSAIDs can have similar effects and fewer side effects compared to oral NSAIDs. These topical agents should be used 4 times a day for 4 weeks. In addition to the findings of clinic trials on the use of NSAIDs in osteoarthritis, these drugs are also effective in the treatment of TMJ inflammation due to orthopathy. The other medications that are used in the management of TMD are tricyclic anti-depressants that can relieve facial joints pain caused by TMJ [52].

TMJ pain can be blocked temporarily by local infusion of anesthetic agents including lidocaine (1% or 2%) in the joint space. The pain resolves after 10 minutes. This is more effective in improving intra-articular pain. Injection site is in the upper joint space and below the straight line from the tragus to the canthus of the eye [53].

Intra-articular steroid injection can be effective in TMJ synovitis. This method is not recommended, unless joint inflammation is confirmed by MRI scan or during arthroscopy. The excessive use of intra-articular steroids may lead to irreversible removal of cartilage. Myofascial pain and spasm can be temporarily relieved by injection of a local anesthetic like bupivacaine 0.5% or botulinum toxin, into the temporalis muscle mass. Measuring pain using a visual analog scale shows that this treatment reduces at least 25% in 79% of the pain. In recent years, Methotrexate and other agents like Hyaluronate have also been considered in the management of TMD in addition to NSAIDs and local anesthetics. In the next sections, the findings of studies that used these agents for treating TMDs will be discussed [54],[55].

Anticonvulsants have been used in some studies for the treatment of TMJ. In the study by Kimos et al., daily administration of 300 mg Gabapentin significantly decreased pain compared to control group [56]. Clonazepam (0.25 mg per night) and diazepam (0.5 mg per day, four times each day) were also found to reduce pain in TMD patients [57],[58]. In the study by DeNucci et al., Triazolam at a dose of 0.125 mg per night, was reported to have no significant effect in reducing pain in TMD patients [59].

### 3.6. Autoimmune diseases that are related to TMD

Autoimmune disease occurs when the immune system erroneously attacks body organs [60]. Autoimmune diseases can involve tissues and organs including red blood cells, blood vessels, thyroid gland, pancreas, muscles, joints and skin [61]. Rheumatoid arthritis is among the autoimmune diseases of the joints, where joints are attacked by the immune system. The immune system attack results in redness, warmth, pain and rigidity in joints. Unlike the senile swelling of joints, that increases with increasing age, RA may occur from the age thirty [62],[63]. In some autoimmune diseases, including psoriasis or RA, the symptoms are not durable and may disappear after a while. The joints and the surrounding tissues are affected in RA. Physicians believe that many systemic conditions including lupus erythematosus, RA and systemic sclerosis are commonly accompanied with TMD [64],[65].

### 3.7. Treatment of TMD accompanied by autoimmune diseases

Rupareila et al. published a short report describing an Indian woman who was diagnosed with TMD and RA. They found that early diagnosis of TMD could help in early diagnosis of other conditions including RA. The reported case received NSAIDs and corticosteroids [67].

Sodhi et al. published a case report describing a 22-year-old patient with the complaint of pain in the left side of the jaw. The patient was diagnosed with RA. TMD was observed in TMJ examinations. They reported that TMD was present in RA with a common radiographic injury in hand and foot joints. Treatment was performed by administration of 75 mg methotrexate [68].

Chebbi et al. reported a case of systemic sclerosis, an autoimmune disease, with hip joint involvement in a 45-year-old woman. The patient was also diagnosed to have TMD. They mentioned that TMD should be suspected by physicians in systemic sclerosis patients. Patients should be thoroughly examined since the effect and discomfort might be limited to jaw, which will result in pain. Furthermore, the treatment is harder if the TMD complications were diagnosed at later stages [69].

Cordeiro et al. assessed the TMD in TMJ of patients with RA. They performed physical assessment of TMJ to categorize TMD in 49 patients of both genders in all age groups. The findings revealed that 75% of patients complained from orofacial pain, and one or both of the following symptoms; arthralgia and myalgia. Degenerative changes were observed in 90% of patients. In order to reduce functional and structural damage, early diagnosis of TMD was recommended for RA patients [70].

**Table 1.** The findings of articles regarding the autoimmune diseases that influence TMD are discusses in this section.

Author	Country	Findings	Reference
Aiko et al. (2011)	Albania	Difficulty and limitation in opening the mouth and pain in TMJ were seen in majority of systemic sclerosis but were observed in only one percent of cases of RA. Treatment was performed with NSAIDs, DMARDs and oral steroids.	[66]
Rupareli et al. (2014)	India	TMD disorders can be diagnosed earlier based on RA related symptoms. Treatment was performed by administration of NSAIDs and corticosteroids.	[67]
Sodhi et al. (2015)	India	TMD occurs in some cases of RA. Treatment was performed by administration of 75 mg methotrexate.	[68]
Chebbi et al. (2016)	Tunisia	Early diagnosis of TMD is required in systemic sclerosis patients. Treatment was performed using conservative methods including rest, reassurance and jaw opening exercises as well as myorelaxants.	[69]
Cordeiro et al. (2016)	Brazil	Diagnosis of TMD in RA patients is necessary in order to reduce structural and functional damages. Treatment was not mentioned in this article.	[70]
Wang et al. (2017)	Taiwan	CIA is a suitable technique for assessment of RA related TMD. Treatment was not mentioned in this article.	[71]
Crincoli et al. (2019)	Italy	The observations revealed that TMD was present in RA patients. Treatment was performed by the administration of corticosteroid, DMARDs and biologic DMARDs.	[72]
Hutami et al. (2019)	Japan	TMJ has specific characteristic that distinguishes it from other joints. Therefore, there is a need for specific treatments for TMD. Blocking nuclear translocation of the p50 NF-κB subunit is a potential treatment option.	[73]
Shi et al. (2003)	China	Hyaluronate was not significantly different from corticosteroids in the reduction of pain and other symptoms. Hyaluronate had potential effects in improving the arthroscopic assessment scores. Transient and mild complications, including discomfort or pain in the injection site, were reported in the hyaluronate group.	[74]
Didem O. Ince et al.	USA	The results of this study revealed that treatment with methotrexate minimized joint destruction in TMD among rheumatoid arthritis patients.	[75]
Sigvard Kopp et al.	Sweden	This study revealed that systemic treatment with combination of infliximab and methotrexate resulted in reduced TMJ related pain in patents with RA, which was correlated with increased cytokine levels and antidepressant receptors in the synovial fluid and plasma.	[76]

Crincoli et al. assessed that prevalence of signs and symptoms of TMD and TMJ and their complications in 52 patients with RA. They found that TMD was present in RA patients. Significant reduction in the saliva production was observed in patients with ERA. Pain in palpation of MP joints was more prevalent and sever in the control group compared to the case group. Treatment was performed by administration of corticosteroids, DMARDs and biologic DMARDs [72].

Shi et al. performed a review study to compare the therapeutic effects of hyaluronate with corticosteroids in TMD. They reported no significant difference in terms of reduction in pain and other symptoms between the two treatments. They reported heterogeneity in the comparison of arthroscopy with arthrocentesis with or without hyaluronate. Hyaluronate was reported to have potential effects in the improvement of arthroscopy assessment scores. Complications were mild and transient and included pain or discomfort in injection site in hyaluronate group. No significant improvement in quality of life was observed in hyaluronate group [74].

Didem O. Ince et al. assessed the effectiveness of methotrexate treatment on TMD and facial morphology in patients with juvenile rheumatoid arthritis. They found that methotrexate administration might minimize destruction in TMD in patients with juvenile rheumatoid arthritis. They also reported that the extent of TMJ involvement in patients with polyarticular form of juvenile rheumatoid arthritis was significantly reduced in methotrexate group compared to control group, who did not receive methotrexate [75]. This study assessed the effects of methotrexate on TMD in patients with severe juvenile rheumatoid arthritis in regards to the side effects of methotrexate.

Beside the studies that assessed the administration of methotrexate in improving TMD symptoms, further studies assessed the combination of methotrexate and other medications in order to improve treatment outcomes.

Sigvard Kopp et al. assessed the effect of administration of methotrexate and infliximab in reduction of temporomandibular joint pain in patients with RA. They assessed the changes in synovial and plasma cytokines. They found that the combination of systemic infliximab and methotrexate resulted in reduction in TMJ pain in patients with RA, which was correlated with increased cytokines and antidepressant receptors in the synovial fluid and plasma. Infliximab has a very specific inhibitory effect on TNF-α. The binding of infliximab to TNF-α results in the degradation of TNF-α [76]. It can be inferred that the observed anti-inflammatory and analgesic effects of infliximab in this study and other studies is due to the inactivation of TNF-α by infliximab.

## 4. Discussion

The current study included 11 studies comprising of 9 studies on the relationship between RA and TMD and 2 studies on the effects of systemic sclerosis on TMD. In the point of view of physicians, several systemic disorders including lupus erythematosus, RA and systemic sclerosis commonly occur with TMD [64],[65]. The authors of this manuscript believe that the assessment of the relationship between each autoimmune diseases and TMD is essential since the progression of TMJ involvement and its negative consequences can be prevented earlier.

In the study by Aliko et al. TMD was assessed in patients with lupus erythematosus and systemic sclerosis [66]. Jonsson et al. reported that TMD is common in lupus erythematosus patients [77]. Lupus erythematosus is a chronic autoimmune disease that affects various organs including joints [78]. Therefore, TMD assessment should be performed for all patients with lupus erythematosus in rheumatology clinics.

Chebbi et al. assessed the effect of systemic sclerosis on TMD. They reported that early diagnosis of TMD in systemic sclerosis patients is necessary [69]. In the study by Mofid et al. the relationship between systemic sclerosis and periodontal and other oral and dental hygiene indices were assessed. They observed that assessment and knowledge on the oral and dental changes due to scleroderma are necessary for dentists because they may lead to earlier diagnosis of systemic sclerosis and reduce disabilities of the patients in performing daily tasks including swallowing, speaking and reducing the unpleasant sense of dryness in mouth [79]. Systemic sclerosis, also called scleroderma, is a multisystem connective tissue disease with an unknown etiology, which is defined by infection, fibrotic and vascular changes in skin and internal organs [80]. Systemic sclerosis has various oral manifestations, including difficulty in mouth opening, telangiectasia of the oral mucosa, xerostomia, thickening of the periodontal ligament and mandibular bone resorption [80].

The mentioned treatments for TMD in the studied articles included oral steroids, DMARDs and NSAIDs, 75 mg methotrexate and combination therapy with methotrexate and administration of hyaluronate. Aiko et al. [66] and Ruparelia et al. [67] used NSAIDs for treatment. NSAIDs, including ibuprofen or naproxen that are prescribed to control pain and inflammation, were the most common NSAIDs used to reduce pain in TMD. Administration of NSAIDs is considered in TMD due to their analgesic and anti-inflammatory effects. Despite the considerable use of NSAIDs in TMD, no RCT has yet been conducted to assess the effects of oral NSAIDs [81].

## 5. Conclusions

The findings of this review revealed that TMD exists in some autoimmune diseases, including RA, lupus erythematosus and systemic sclerosis. Based on majority of the studied articles early diagnosis of TMD results in the prevention of progression in the primary stages and reduce treatment costs. Therefore, it is necessary to identify the prevalence of TMD in different regions and to identify the influencing factors related to TMD. Furthermore, medications including oral steroids, DMARDs, NSAIDs and methotrexate (75 mg) were used in the studied articles in this review. Combination therapy, including combination of methotrexate and other anti-inflammatory medications, is being used recently in the management of TMD. It seems that multiple treatments that implement the combination of different medications and physical methods, including physiotherapy and etc., can improve treatment outcomes. Regarding the findings of this review, as the prevalence of TMD in autoimmune diseases was not identified, it is recommended to conduct further research regarding the prevalence of TMD and its relationship with autoimmune diseases, geographical regions, lifestyle and nutritional habits. Furthermore, there is a need to assess TMD in different age groups especially among adolescents, as this age group is susceptible to TMD. Lack of knowledge on the risk factors and prevalence of TMD is due to the absence of a clear definition, and lack of clinical and history criteria for TMD. Due to the complications of TMD, there is a need to establish a data base by the ministry of health regarding patients with TMD in order to improve patient follow up by dentists and to be able to assess the effectiveness of primary treatment and TMD medications in order to choose a more suitable treatment modality. It is also recommended for physicians to have interdisciplinary collaboration with dentists to assess autoimmune diseases to prevent the pain caused by these conditions.
